# Preoperative risk factors for extended hospital stay: A prospective study in a South African clinic

**DOI:** 10.4102/phcfm.v17i1.4781

**Published:** 2025-03-20

**Authors:** Kuven Naidu, Nabeela Kajee, Jayseelan Naidu, Bilaal Wadee

**Affiliations:** 1 East Rand Physicians, Faculty of Medicine, Benoni, South Africa

**Keywords:** preoperative medicine, extended length of stay, risk stratification, comorbidities, hospital resource utilisation, surgical outcomes

## Abstract

**Background:**

Preoperative assessment clinics play a critical role in identifying, evaluating and mitigating perioperative risks. Despite global data highlighting the importance of preoperative risk factors on surgical outcomes, there remains limited information on their impact on post-operative length of stay (LOS) in South African contexts.

**Aim:**

This study aimed to describe the demographic and clinical profiles of patients referred to a preoperative clinic as well as factors associated with post-operative extended LOS.

**Setting:**

The preoperative clinic is based in the city of Johannesburg in South Africa.

**Methods:**

This was a prospective cohort study conducted between 2021 and 2022 at a private clinic in patients undergoing non-cardiac surgery. Data on demographics, co-morbidities, surgical procedures and clinical outcomes were collected. Statistical analysis was performed to assess relationships between preoperative risk factors, including ASA grading, Revised Cardiac Risk Index (RCRI), estimated glomerular filtration rate, diabetes mellitus, age, obesity and LOS.

**Results:**

A total of 214 patients were assessed, of which 75.7% were female, with a median age of 62.5 years. Common co-morbidities included hypertension (59.3%) and obesity (55%). The median LOS was 3.5 days, with 47.2% of patients staying more than 3 days post-operatively. Knee (33.2%) and hip surgeries (21%) were the most common procedures. A significant association was found between longer LOS and RCRI score ≥ 1 (*p* = 0.007), renal dysfunction in knee surgery patients (*p* = 0.027) and age in patients undergoing hip surgery (*p* = 0.049).

**Conclusion:**

Findings note the need for targeted intereventions in preoperative care to reduce LOS, particularly for high-risk patients.

**Contribution:**

This study highlights the fact that preoperative information may play a significant role in patient’s outcomes post surgery. Further research is needed to validate these results across broader healthcare settings.

## Introduction

Preoperative assessment is an integral aspect of surgical care, offering a unique opportunity to identify, evaluate and mitigate perioperative risks. The effectiveness of a preoperative clinic can dramatically affect patient outcomes and healthcare resource allocation. With over 234 million major non-cardiac surgeries performed globally each year, perioperative complications, including major adverse cardiac events (MACE), significantly contribute to patient morbidity, mortality and prolonged length of stay (LOS).^1^ Preoperative clinics play a critical role in identifying medical issues, either new or old, that may impact on a patients surgical journey.^2^ It has been established that preoperative risk factors are a significant predictor of increasing hospital costs more so than actual procedure complications themselves.^3^ Preoperative clinics have been shown to decrease LOS^4,5^ and play important roles in decreasing unnecessary and unwarranted consultations.^6^ Patients who attended a preoperative clinic were also less likely to have their procedures cancelled on the day of admission for the procedure by an anaesthetist.^5^ This can be a source of frustration for the patient, the doctor and the facility.

Prolonged LOS increases the cost per admission. A South African study has estimated the cost per day in intensive care unit (ICU) to be approximately R22 870 per day in the public sector.^7^ A day in the surgical general ward would cost approximately R4978 per day.^8^ It will therefore be important to identify any possible causes beforehand that may affect LOS in hospital post procedure. The revised cardiac risk index (RCRI) score can identify patients undergoing noncardiac surgery in whom complications are more likely to occur.^9^ A modified risk score of greater than or equal to 3 has also been shown to have increased non-cardiac morbidity as well as a prolonged hospital stay after elective orthopaedic procedures.^10^ However, there remain limited South African data on the value of using an RCRI as a risk factor for prolonged stay post-surgery. The American Society of Anesthesiologists (ASA) Grading system (I–VI) is used to determine the health of a patient before undergoing a procedure.^11^ A prolonged LOS has also been noted with an increase from ASA Class I to ASA Class IV.^12^ Co-morbidities may also play an important role in prolonging LOS post procedure. Age,^13^ diabetes mellitus,^14^ hypertension,^15^ obesity,^16^ anaemia^17^ and impaired renal function^18^ have all been noted to play a role in extending LOS postoperatively.

The objectives of this study were to describe the demographic and clinical profile of patients referred for a preoperative assessment to a private clinic in the East Rand of Johannesburg. We looked at the post-operative LOS in this cohort of South African patients and possible associated risk factors. This will add to the currently limited data available for South African centres hopefully allowing for further insight into risk stratification and appropriate resource planning.

## Research methods and design

### Study design, study setting and study population

This was a prospective cohort study. The cohort comprised patients enrolled at the preoperative clinic in a private practice in Benoni, Johannesburg between 25 Jan 2021 and 15 June 2022. These patients were referred by private surgeons to obtain a preoperative assessment prior to being admitted for elective surgery.

Inclusion criteria were any patient over the age of 18 years referred for non-cardiac surgery to the preoperative clinic and who were willing and able to give consent.

All patients who attended the clinic were approached to participate. The preoperative assessment that was performed at the initial visit formed part of the data collection.

There were no specific exclusion criteria other than patients under the age of 18 years and those unwilling to participate.

Data sources were the preoperative assessment notes, and LOS was provided by the hospital to which the patients were admitted. A request was made via email to the hospital the patient was admitted to, and admission details were provided via return email. Patient data were limited to date of discharge post procedure.

Data on demographic characteristics (age, biological sex, race) and clinical characteristics (body mass index [BMI], co-morbidities, ASA grading, RCRI index), haematological (haemoglobin and haematocrit,) and clinical biochemistry parameters (urea, creatinine, glomerular filtration rate, sodium, potassium, chloride and random blood glucose), type of surgical procedure planned, ICU or high-care admission as well as length of hospital stay post-operatively were extracted from the notes and hospital records.

### Data collection and analysis

Data from the medical records were captured in a study specific Excel database. Data were exported into Stata 18.0 [Stata Corporation, College Station, USA] for analysis.

Data set available online for review at DOI: 10.5061/dryad.sbcc2frh1.

Demographics (age, sex, race, date of assessment), clinical co-morbidities (hypertension, diabetes mellitus, dyslipidaemia, hypothyroidism, asthma, chronic obstructive pulmonary disease and other co-morbidities), haematological and biochemical results of included participants were described using categorical and continuous variables. Categorical variables (sex, race, co-morbidities) were described as frequencies and percentages. Continuous variables (age, haematological and biochemical results), which are normally distributed, were described as means and standard deviations while variables that are not normally distributed were described as medians with interquartile ranges. Appropriate graphs and charts were used to visualise the data.

The primary outcome of the study was the length of hospital stay. This was determined as the number of days from admission to discharge and was presented as a median and interquartile range. The length of hospital stay was described and presented overall and based on the type of surgical procedure indicated, ASA grading, age, BMI category and by presence of renal dysfunction, anaemia or diabetes. The *X*^2^ test, K-test for equality of medians or the robust test of equal variance (for normally distributed data) were used to determine whether the demographic and clinical characteristics of patients who stayed in hospital for ≥ 1 day differed by type of surgical procedure.

Univariable and multivariable Poisson regression with robust error variance was used to determine the demographic and clinical factors that were independently associated with post-operative admission to ICU or high care. Variables that had a *p*-value < 0.2 in univariable analyses were included in the multivariable model with age, sex, ASA grading and type of surgical procedure included *a priori*. The strength of association between the different factors and high care or ICU stay post-operatively was determined as a relative risk (RR) with a 95% confidence interval around it.

Another univariable and multivariable Poisson regression model with robust error variance was used to determine factors independently associated with length of hospital stay > 3 days post-operatively. Variables that had a *p*-value < 0.2 in univariable analyses were included in the multivariable model with age, sex, ASA grading and type of surgical procedure included in the multivariable model a *priori*. The strength of association between the different factors and high care or ICU stay post-operatively was determined as a RR with a 95% confidence interval around it.

### Sample size considerations

There was no sampling or selection of patients for inclusion in the study. Rather all eligible patients who gave consent were included in the analysis. A post hoc power calculation was conducted to determine power for the realised sample size.

### Ethical considerations

Ethical clearance to conduct this study was obtained from the Life Glynnwood Hospital, National Health Research Ethics Committee of Life Healthcare Group (No. REC 251015-048).

## Results

A total of 214 patients were seen at the preoperative clinic with a total of 213 cleared to proceed with surgery (Table 1). Seven patients of the 213 cleared (3.3%) had their procedures or surgeries postponed or cancelled prior to undergoing surgery. These cancellations comprised patients deciding to forego the planned procedure. Of the 214 seen in the preoperative clinic and operated on, 152 (71.0%) were admitted into the hospital for 1 day or longer. The sample size of 214 had a power of 98% to determine an admission rate of 71% ± 6.5 days assuming an α-level of 0.05.

**TABLE 1:** Demographic characteristics of patients seen at the preop clinic (*N* = 214).

Variable	*n*	%	Median	IQR
Age			62.5	53–71
**Sex**				
Male	52	24.3	-	-
Female	162	75.7	-	-
**Race (self-identifying)**				
Black African	22	10.3	-	-
White	176	82.2	-	-
Indian	16	7.5	-	-
Coloured	0	0.0	-	-
**Year of enrolment**				
2021	154	72.0	-	-
2022	60	28.0	-	-

IQR, interquartile range.

The mean age of the patients was 61.4 years (median of 62.5 years). The majority of the patients were female (75.7%). The majority of the patients self-identified as white (82.2%) with 10.3% identifying as Black African and 10.3% as Indian.

**FIGURE 1:**
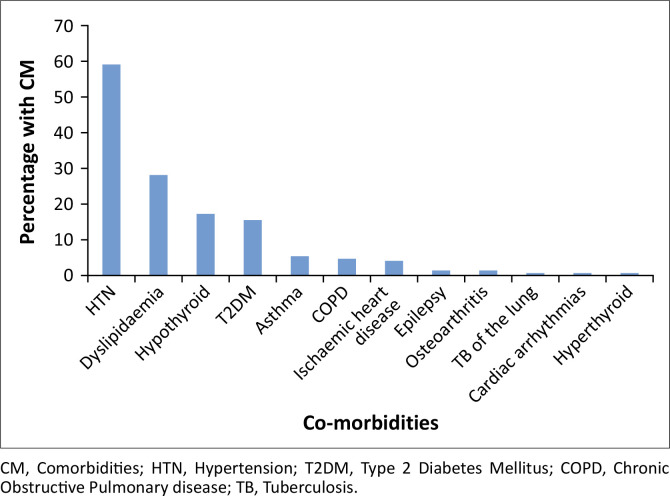
Frequency of co-morbidities preoperatively (*N* = 214).

The four most frequent co-morbidities were hypertension (*n* = 127; 59.3%), dyslipidaemia (*n* = 61; 28.5%), hypothyroidism (*n* = 38; 17.8%) and Type 2 diabetes mellitus (*n* = 30; 14%) (Figure 1).

The majority of the patients were referred for elective knee surgery (*n* = 71; 33.2%), hip surgery (*n* = 45; 21%), hysterectomy or hysteroscopy and (*n* = 43; 20%) and breast surgery (*n* = 19; 8.9%).

There was BMI data available in 211 of the patients with 55% being classified as obese (BMI > 30 kg/m^2^).

The median blood pressure reading in the patients was 134/80.5 mmHg with 30.8% having a systolic blood pressure reading over 140 mmHg and 11.2% having a diastolic reading above 90 mmHg (Table 2).

**TABLE 2:** Clinical profile of patients seen in the preoperative clinic (*N* = 214).

Parameter	*N*	Median	IQR	Abnormal*	
				*n*	%
BMI (m/kg^2)^	211	30.9	27–35.2	116	55.0
SBP (mmHg)	214	134	124–143	66	30.8
DBP (mmHg)	214	80.5	74–85	24	11.2

IQR, interquartile range; BMI, body mass index; SBP, systolic blood pressure; DBP, diastolic blood pressure.

* Abnormal for SBP > 140mmHg; Abnormal for DBP > 90mmHg.

The baseline biochemical profile of the patients is listed in Table 3.

**TABLE 3:** Biochemical profile of patients seen in the preoperative clinic, *N* = 214.

Parameter	*N*	Median	IQR	Abnormal	
				*n*	%
Haemoglobin (g/dL)	205	14.3	13.3–15.3	41	20.0
Haematocrit (L/L)	201	40	28–44	5	2.5
Sodium (mmol/l)	203	140	138–141	19	9.4
Potassium (mmol/L)	203	4.2	3.9–4.4	15	7.4
Chloride (mmol/L)	202	103	101–106	33	16.3
Urea (mmol/L)	205	5.7	4.4–6.8	39	19.0
Creatinine (umol/L)	211	72	62–84	46	21.8
GFR (mL/min)	204	83.06	70.08–90.76	23	11.3
Random blood glucose (mmol/L)	207	5.4	5.0–6.2	12	5.8

Note: Normal values: Hb males 13.8 g/dL – 18.8 g/dL, Hb females 12.4 g/dL – 16.7 g/dL; HCT males 0.4 L/L – 0.56 L/L, HCT females 0.35 L/L – 0.49 L/L females; Sodium 136 mmol/L – 145 mmol/L; Potassium 3.5 mmol/L – 5.1 mmol/L; Chloride 98 mmol/L – 107 mmol/L; Urea 2.1 mmol/L – 7.1 mmol/L; Creatinine males 80 umol/L – 115 umol/L, Creatinine females 53 umol/L – 97 umol/L; GFR > 60 mL/min; Random blood glucose 4.0 mmol/L – 11.1 mmol/L.

IQR, interquartile range; GFR, glomerular filtration rate.

The majority of the patients seen were graded as an ASA Grade 2 (81.2%) with those graded as an ASA 1 or 3 being 5.6% and 13.2%, respectively (Online Appendix Figure 1-A1). A total of 74.5% of patients had an RCRI score of 0, 23.8% a score of 1 and 1.4% a score of 2. No patients were noted to have an RCRI score of 3 in the cohort (Online Appendix Figure 2-A1).

Table 4 highlights the characteristics of patients who were admitted for longer than a day in hospital. A total of 152 (71%) patients stayed for 1 day or longer. No significant differences were noted between the different surgical procedures other than patients undergoing uterine surgery who had a larger proportion of patients (48.4%) with an RCRI score of 1 or 2.

**TABLE 4:** Characteristics of patients who stayed in hospital for one day or more by planned surgical procedure (*N* = 152).

Variable	Other (*n* = 23)						Knee (*n* = 47)						Hip (*n* = 34)						Breast (*n* = 17)						Uterine (*n* = 31)						*p*
	*n*	%	Mean	s.d.	Mean	%	*n*	%	Mean	s.d.	Mean	%	*n*	%	Mean	s.d.	Mean	%	*n*	%	Mean	s.d.	Mean	%	*n*	%	Mean	s.d.	Mean	%	
Age	-	-	60.3	14.1	-	-	-	-	63.7	10.3	-	-	-	-	63.2	10.7	-	-	-	-	67.5	10.4	-	-	-	-	51.9	10.3	-	-	0.267*
Male	3	13.0	-	-	-	-	15	31.9	-	-	-	-	17	50.0	-	-	-	-	0	0	-	-	-	-	0	0	-	-	-	-	….
BMI kg/m^2^	-	-	-	-	29.2	7.2	-	-	-	-	33.1	5.7	-	-	-	-	29.5	6.1	-	-	-	-	32.4	8.2	-	-	-	-	31.9	7.2	0.361*
BMI greater than 30 kg/m^2^	10	43.4	-	-	-	-	31	66.0	-	-	-	-	14	41.2	-	-	-	-	8	47.1	-	-	-	-	17	56.7	-	-	-	-	0.177
RCRI score 1/2	6	26.1	-	-	-	-	8	17.0	-	-	-	-	7	20.6	-	-	-	-	3	17.7	-	-	-	-	15	48.4	-	-	-	-	0.024
Hb g/dL	-	-	13.6	1.7	-	-	-	-	14.7	1.8	-	-	-	-	14.6	1.8	-	-	-	-	14.2	1.9	-	-	-	-	13.4	2.9	-	-	0.081
Low HB	6	26.1	-	-	-	-	4	8.5	-	-	-	-	5	14.7	-	-	-	-	3	17.7	-	-	-	-	8	25.8	-	-	-	-	0.204µ
eGFR	-	-	79.9	20.3	-	-	-	-	79.0	18.7	-	-	-	-	79.9	17.3	-	-	-	-	86.5	22.1	-	-	-	-	86.6	11.3	-	-	0.436*
GFR < 60	4	17.4	-	-	-	-	8	17.0	-	-	-	-	2	6.1	-	-	-	-	2	12.5	-	-	-	-	1	3.5	-	-	-	-	0.268µ
Diabetes mellitus	5	21.7	-	-	-	-	6	12.8	-	-	-	-	4	11.8	-	-	-	-	4	23.5	-	-	-	-	3	9.7	-	-	-	-	0.559
Hypertension	16	69.6	-	-	-	-	29	61.7	-	-	-	-	18	52.9	-	-	-	-	10	58.8	-	-	-	-	15	48.4	-	-	-	-	0.547
Other co-morbidities	5	21.7	-	-	-	-	7	14.9	-	-	-	-	8	23.5	-	-	-	-	5	29.4	-	-	-	-	7	22.6	-	-	-	-	0.743

Note: The K-test for equality of medians is used to test the hypothesis that the medians across multiple groups are equal.

s.d., standard deviation; BMI, body mass index; RCRI, revised cardiac risk index; GFR, glomerular filtration rate; eGFR, estimated glomerular filtration rate; Hb, haemoglobin.

* , *p*-value for robust test of equal variance; µ, *p*-value for Fisher’s exact test.

### Results – Length of stay

A total of 152 patients (71%) stayed in hospital for more than a day or longer, 15 were admitted into an ICU and 99 into a high care unit (HCU). The median LOS was 3.5 days (interquartile range [IQR] 2.8–4.5 days). The median LOS for knee surgery, hip surgery, breast surgery and uterine surgery was 3.5, 4.0, 3.0 and 3.5 days, respectively (Online Appendix Figure 3-A1). A total of a 101 (47.2%) patients were admitted for more than 3 days (Online Appendix Figure 4-A1).

The orthopaedic procedures (hip [*p* = 0.001] and knee surgery [*p* = 0.039]) were more likely to be admitted to ICU or high care than either breast or uterine surgical procedures (Table 5).

**TABLE 5:** Factors associated with admission to intensive care unit or high care post-surgery among patients seen in the preoperative clinic.

Variable	% admitted to ICU/high care post-op		Univariable RR	95% CI	*p*	Multivariable RR	95% CI	*p*
	*n*	%						
Age (years)	-	-	1.01	1.00–1.02	0.055	1.00	0.99–1.02	0.808
**Sex**								
Female	67/162	41.4	1.00	-	-	1.00	-	-
Male	32/52	61.5	1.49	1.12–1.97	0.006	1.01	0.75–1.34	0.970
**Race**								
Other	16/38	42.1	1.00	-	-	-	-	-
White	83/176	47.2	1.12	0.75–1.68	0.584	-	-	-
**Year**				1.10–2.45				
2021	80/154	52.0	1.64	-	0.016	1.57	1.06–2.31	0.023
2022	19/60	31.7	1.00	-	-	1.00	-	-
**BMI**	-	0.99	0.97–1.01	-	0.539	-	-	-
**One or more co-morbidities**								
No	20/42	47.6	1.00	-	-	-	-	-
Yes	79/172	45.9	0.96	0.67–1.38	0.843	-	-	-
**ASA grading**								
1	6/12	50.0	1.27	0.61–2.64	0.518	1.06	0.53–2.15	0.866
2	81/173	46.8	1.19	0.73–1.94	0.481	1.23	0.76–1.99	0.401
3	11/28	39.3	1.00	-	-	1.00	-	-
**RCRI points**								
0	71/161	44.1	1.00	-	-	1.00	-	-
1/2	28/53	52.8	1.20	0.88–1.63	0.252	1.33	0.99–1.78	0.054
**eGFR**	-	0.99	0.983–0.998	-	0.017	1.00	0.988–1.00	0.199.
**Hypertension**								
No	40/88	45.5	1.00	-	-	-	-	-
Yes	59/126	46.8	1.03	0.77–1.38	0.844	-	-	-
**Diabetes mellitus**								
No	83/180	46.1	1.00	-	-	-	-	-
Yes	16/34	47.1	1.02	0.69–1.51	0.919	-	-	-
**Planned procedure**								
Other	11/34	32.4	1.00	-	-	1.00	-	-
Knee surgery	39/71	54.9	1.70	1.00–2.89	0.051	1.74	1.03–2.95	0.039
Hip surgery	32/45	71.1	2.20	1.30–3.70	0.003	2.38	1.39–4.05	0.001
Breast	6/21	28.6	0.88	0.38–2.03	0.770	0.97	0.42–2.25	0.940
Uterine	11/43	25.6	0.79	0.39–1.60	0.514	0.66	0.31–1.37	0.264

ICU, intensive care unit; RR, relative risk; CI, confidence interval; BMI, body mass index; ASA, American Society of Anesthesiologists; RCRI, Revised Cardiac Risk Index; eGFR, estimated glomerular filtration rate.

There was a trend towards admitting patients with a RCRI score of 1 or 2 to ICU or high care, but it did not reach significance (*p* = 0.054).

There was no association between preoperative ASA grading and utilisation of high care or ICU postoperatively (ASA 1 [*p* = 0.866], ASA 2 [0.401]).

Females were more likely to be admitted for longer than 3 days (Table 6) as compared to their male counterparts (*p* = 0.003).

**TABLE 6:** Factors associated with admission for > 3 days’ post-surgery among patients seen in the preoperative clinic.

Variable	% admitted to hospital > 3 days		Univariable RR	95% CI	*p*	Multivariable RR	95% CI	*p*
	*n*	%						
Age (years)			1.00	0.99–1.01	0.953	1.00	0.99–1.01	0.943
**Sex**								
Female	81/162	50.0	1.30	0.89–1.90	-	1.73	1.20–2.48	0.003
Male	20/52	38.5	1.00	-	0.173	1.00	-	-
**Race**								
Other	23/38	60.5	1.37	1.01–1.86	0.046	1.62	1.19–2.21	0.002
White	78/176	44.3	1.00	-	-	1.00	-	-
**Year**								
2021	78/154	50.7	1.32	0.92–1.89	0.127	1.23	0.87–1.74	0.252
2022	23/60	38.3	1.00	-	-	1.00	-	-
**BMI**	-	0.99	0.97–1.01	-	0.338	-	-	-
**One or more co-morbidities**								
No	20/42	47.6	0.951	-	-	-	-	-
Yes	81/172	47.1	0.99	0.69–1.49	0.951	-	-	-
**ASA grading**								
1	6/12	50.0	1.27	0.61–2.64	0.518	1.03	0.50–2.13	0.940
2	83/173	48.0	1.22	0.75–1.99	0.421	1.20	0.74–1.95	0.459
3	11/38	39.3	1.00	-	-	1.00	-	-
**RCRI points**								
0	68/161	42.2	1.00	-	-	1.00	-	-
1/2	33/53	62.3	1.47	1.12–1.95	0.006	1.52	1.12–2.07	0.007
**eGFR**	-	0.99	0.98–1.00	-	0.032	0.99	0.98–1.00	0.031
**Hypertension**								
No	44/88	50.0	1.11	0.83–1.47	0.491	-	-	-
Yes	57/126	45.2	1.00	-	-	-	-	-
**Diabetes mellitus**								
No	89/180	49.4	1.40	0.87–2.26	0.168	1.60	1.01–2.52	0.044
Yes	12/34	35.3	1.00	-	-	1.00	-	-
**Planned procedure**								
Other	12/34	35.3	1.00	-	-	1.00	-	-
Knee surgery	32/71	45.1	1.28	0.76–2.16	0.360	1.52	0.95–2.44	0.083
Hip surgery	27/45	60.0	1.7	1.02–2.85	0.043	2.36	1.47–3.80	< 0.001
Breast	8/21	38.1	1.08	0.53–2.20	0.833	1.18	0.60–2.33	0.627
Uterine	22/43	51.2	1.45	0.84–2.49	0.179	1.20	0.70–2.05	0.502

RR, relative risk; CI, confidence interval; BMI, body mass index; ASA, American Society of Anesthesiologists; RCRI, Revised Cardiac Risk Index; eGFR, estimated glomerular filtration rate.

Patients with an RCRI score of 1 or greater were more likely to be admitted for a prolonged period (*p* = 0.07) as were those patients who had undergone knee surgery (*p* ≤ 0.001)

Diabetic patients were less likely to have spent more than 3 days in hospital (*p* = 0.044).

Length of stay per procedure type was assessed against the following risk factors: ASA grade, estimated glomerular filtration rate (eGFR), low Hb (< 13.8 g/dL in males; < 12.4 g/dL in females), obesity (defined as BMI ≥ 30 kg/m^2^) and presence of diabetes mellitus.

There was only a significant association with a low eGFR in knee surgery (*p* = 0.027) and age in hip surgery (*p* = 0.049) (Table 7).

**TABLE 7:** Length of stay per procedure and associated possible risk factors.

Risk factors	Knee surgery			Hip surgery			Breast surgery			Uterine		
	Median	IQR	*n*	Median	IQR	*n*	Median	IQR	*n*	Median	IQR	*n*
**Length of stay by ASA grading and procedure**												
ASA grading												
1	3.5	3.5–5.5	3	4	3.25–4.75	4	-	5	5–5	1	-	-
2	3.5	3–4	38	4	3.5–5.5	26	3	2–3.5	15	3.5	3–4.25	24
3	3	2.5–4	5	5	3.5–7	4	2.25	1–3.5	2	3.5	3.5–4.5	6
p**	0.317	-	-	0.767	-	-	0.929	-	-	0.434	-	-
**Length of stay by renal dysfunction and procedure**												
GFR												
GFR < 60	4	3.5–6.25	8	3.75	2.5–5	2	4	3–5	2	6.5	6.5–6.5	1
GFR ≥ 60	3.5	3–4	39	4	3.5–5.5	31	3	2–3.5	14	3.5	3–4.25	28
p*	0.027	-	-	0.640	-	-	0.550	-	-	0.138	-	-
**Length of stay by anaemia and procedure**												
Hb												
Normal Hb	3.5	3–4	43	3.5	3–4	29	2.8	2–3.5	14	3.5	3–4.5	23
Low Hb	3.8	2.75–4	4	5.5	5–8	5	3.5	1–5.5	3	3.5	2.5–4.25	8
p***	0.848	-	-	0.103	-	-	0.762	-	-	0.662	-	-
**Length of stay by BMI and procedure**												
BMI category												
< 30	3.5	2.75–4	16	4	3.5–5.5	20	2.5	2–3.5	9	4	3.5–4.5	13
≥ 30	3.5	3–4	31	4	3.5–5	14	3.3	1.8–4.3	8	3.5	2.5–3.5	17
p*	0.508	-	-	0.923	-	-	0.826	-	-	0.080	-	-
**Length of stay by diabetes and procedure**												
Diabetes												
No	3.5	3–4	41	4	3.5–5.5	30	3.5	2–3.5	13	3.5	3.3–4.5	28
Yes	3.5	3–4	6	5.3	3.5–7	4	2.5	1.5–4	4	3	1.5–3.5	3
p*	0.650	-	-	0.336	-	-	0.577	-	-	0.148	-	-
**Length of stay by age category and procedure**												
Age (years)												
< 50	3.5	3–3.5	5	3.8	3–4	4	5.5	5.5–5.5	1	3.5	2–4	14
50–65	3.5	2.5–3.75	16	4	3.5–5	17	3.5	3.5–3.5	5	3.5	3.5–4.5	12
> 65	4	3.5–5	26	4.5	3.5–6	13	2.5	2–3.5	11	3.5	3–6	5
p**	0.111	-	-	0.049	-	-	0.081	-	-	0.951	-	-

IQR, interquartile range; BMI, body mass index; ASA, American Society of Anesthesiologists; GFR, glomerular filtration rate; Hb, haemoglobin.

*, Ranksum test; **, K-test for equality of medians; ***, Ranksum test; Low Hb was defined as: Hb males 13.8 g/dL – 18.8 g/dL, Hb females 12.4 g/dL – 16.7 g/dL.

## Discussion

### Revised cardiac risk index score

The association between LOS and an increased RCRI score in our population is in keeping with previous studies in which it was noted that there was an increase in LOS in patients with an increased RCRI score.^19,20^ This could be because of the fact that these patients require closer monitoring because of their increased risk factors. We did not show a significant increase in ICU or High care usage according to the RCRI in our sample; however, there was a signal that trended towards significance. Patients with increased risk factors may also have delayed recovery and rehabilitation may be a more gradual process as healthcare practitioners may show increased caution in dealing with these particular patients.

### American Society of Anaesthesiologist grading

In our study, ASA grading was not associated with an overall prolonged LOS. This is not in keeping with findings from other studies in which there was an increase in length of stage between ASA grade 2 and grade 3.^21^

Furthermore, the need for admission to ICU post op was not influenced by the patients preoperative ASA grading. This was also not in keeping with other studies in which patients with higher preoperative ASA gradings spent a longer period in ICU post op.^22^

A possible explanation for the above findings is that elective surgeries in private tend to be largely protocolised. This results in patients being admitted to ICU or high care regardless of their risk profile and this is an area that will need to be altered based on future guidelines.

### Anaemia

Approximately 25.8% of patients booked for elective uterine surgical procedures were found to be anaemic. This was greater than patients undergoing elective knee (8.5%), hip (14.7%) or breast surgery (17.7%). This is unsurprising as patients undergoing uterine surgery are more likely to have experienced some form of abnormal uterine bleeding resulting in anaemia.^23^ It is somewhat surprising that anaemia did not result in an extended LOS. A study by Wang et al.^17^ in patients undergoing non-cardiac and non-obstetric surgery showed that patients who stayed longer than 7 days had, on average, a lower preop Hb compared to those patients who stayed 7 days or less. Furthermore, a preoperative Hb of less than 11.9 g/dL had a decrease in LOS by 2 days for every 1 g/dL increase in Hb. This was again noted in a study conducted by Bulte et al.,^24^ which highlighted the association of anaemia with LOS with an increase on average of 1.3 days noted in patients with moderate to severe anaemia.

A systemic review and meta-analysis by Fowler et al.^25^ indicated that preoperative anaemia was associated with increased risk of mortality (odds ratio [OR] 2.9), infection (OR 1.93) and acute kidney injury (OR 3.75). All of the above could result in a prolonged LOS.

A possible explanation for why we did not show an increased LOS in patients with a preop anaemia is that once identified patients were provided with iron supplementation and advised to discontinue drugs that situation may worsen iron deficiency (e.g. anti-inflammatories). Minimally invasive surgical techniques may also have prevented significant blood losses in theatre. A form of selection bias was that only patients who were considered fit for surgery and did not require significant optimisation moved forward to having their planned procedures. This resulted in fewer complications and thus no impact on LOS.

### Renal dysfunction

Chronic renal dysfunction was noted to have an impact on morbidity and mortality in patients undergoing surgery.^26^ In particular, Liao et al. showed that patients with renal insufficiency (GFR < 60 mL/min) were hospitalised for a longer period than those without renal insufficiency (3 days vs. 2 days; *p* < 0.0001).^18^

In our study, patients undergoing elective knee surgery with preoperative eGFRs of < 60 mL/min were noted to have an increased LOS, which is in keeping with the literature. There were very few patients undergoing other surgeries who had a decreased eGFR preop, which may have been because of the smaller sample sizes in those groups.

### Obesity

Obesity is a major cause of morbidity and may result in a reduced life expectancy.^27^ It is estimated that currently there are over a billion people worldwide classified as obese (BMI ≥ 30).^28^ A large meta-analysis by Plassmeier et al.^29^ indicated that patients who were obese had longer hospital stays after undergoing colorectal surgery, upper gastrointestinal procedures and pancreatic surgery. Patients who underwent total hip arthroplasty were also noted to have had an increased LOS.^30^ Obesity did not result in an extended LOS in our study. This is in keeping with other studies in which LOS was not impacted by obesity.^31^ In particular, obesity was not associated with an extended LOS following primary hip arthroplasty in a South African setting.^32^ A possible explanation for this is that our study population consisted of patients in a private healthcare setting. These patients generally have increased access to healthcare resources ensuring that co-morbidities are detected and managed earlier. This should lower their overall cardiometabolic risk and decrease subsequent complications post op, which may have resulted in no increase in LOS.

### Type 2 diabetes mellitus

Type 2 diabetes was a co-morbidity in 30 patients (14%). There are multiple studies that report an increase in LOS in diabetic patients compared to non-diabetic patients.^33,34,35,36^ There was no increase in admission to high care or ICU or an increase in LOS noted in patients with type 2 diabetes in our cohort. This is in keeping with a previous study by Lejeune et al. in which type 2 DM did not confer a risk for increased LOS in patients undergoing colo-rectal surgery.^37^ This was attributed to the multi-disciplinary approach to patient care in the peri-operative period. The patients in our cohort were aware of their type 2 diabetic status and were educated in the preop clinic to ensure tight control in the peri-operative period. The increased awareness around the patients Type 2 diabetes status by a multi-disciplinary team may, in our case, also have limited deleterious effects often experienced by these patients in the post-operative period such as post-operative pneumonia^35^ and urinary tract infections.^38^

### Sex

There was a preponderance of females in this cohort. This was likely because of the fact that there were a number of patients referred for pre-assessment of uterine and breast surgery, which will have altered gender pattern in the cohort.

### Age

Age has been identified as a risk factor in predicting an extended LOS post-operatively.^13,39,40^ Ageing is associated with frailty and disability,^41,42^ which will decrease a patients mobility and the ability to withstand a severe stressor such as invasive surgery. Dlamini et al. also showed in their study that a maximum walking distance of less than 100 m resulted in an extended LOS^32^ and the older, frail patient may fall in this category. A previous study looking at outcomes post hip and knee arthroplasty in a New Zealand population noted the LOS generally increased with age except in the age group less than 40 years.^43^

In our study, patients over the age of 65 years who had undergone hip surgery had a prolonged LOS (4.5 days) compared to patients between 50 and 65 years (4 days) and patients under 50 years (3.8 days) [*p*-value 0.049]. There was no difference in LOS based on age in the other three main surgical procedures (knee, uterine, breast).

The average LOS in patients undergoing hip arthroplasty was longer than patients undergoing knee arthroplasty in each of the age ranges (< 50 years; 50–65 years; > 65 years). There was no significant difference in age between the patients in our cohort who had undergone knee and hip arthroplasty (63.7 years vs. 63.2 years, respectively). In the previously mentioned New Zealand study,^43^ 29.9% of patients undergoing a primary hip replacement had a LOS longer than 5 days compared to 34.2% of patients who had undergone a primary knee replacement.

A possible reason for our findings may be procedural in nature. An anterior approach compared to a posterior approach in hip replacements may result in shorter LOS.^44^ However, our study did not include any information regarding type of procedure, which would have allowed us to validate this theory.

## Conclusion

This study provides valuable insights into the demographic and clinical characteristics of patients referred to a preoperative clinic in a private practice in Johannesburg and highlights the factors influencing post-operative LOS. Based on our findings, it may be considered prudent to pay closer attention to a patient’s preoperative RCRI score. The necessity to perform certain preoperative tests may also need to be reviewed as they have had no significant impact on LOS. Any cost-saving initiative is to be explored in South Africa’s socio-economic setting. The trend to performing preoperative blood tests in patients undergoing elective surgery perhaps needs to be re-visited based on our findings. It is also important to establish evidence-based national guidelines tailored to the South African healthcare system. These should include recommendations for preoperative assessments, risk stratification tools and optimisation protocols, ensuring consistency in care across public and private sectors. These guidelines would also help reduce unnecessary costs by avoiding redundant tests and consultations.

### Limitations

This study has several limitations that must be acknowledged. Firstly, the study was conducted in a single private preoperative clinic in Johannesburg, which may limit the generalisability of the findings to other settings, particularly public hospitals or clinics in other regions of South Africa where patient profiles and resource availability may differ.

Secondly, the relatively small sample size, particularly within subgroups such as patients with renal dysfunction or undergoing specific types of surgeries, may limit the statistical power to detect significant associations between certain risk factors and LOS. A larger cohort may have provided more robust results, especially in assessing the impact of variables like ASA grading and RCRI on post-operative outcomes.

Thirdly, the study did not account for the potential influence of surgical techniques, such as anterior versus posterior approaches in hip replacement surgeries, which may affect the LOS. Future studies incorporating procedural differences may provide a clearer understanding of their role in recovery times. The cohort also consisted of patients treated by different surgeons and anaesthetists. Length of stay may differ according to the surgeon’s experience and technique, which could not be commented on.

Fourthly, the study relied on medical records for data collection, which could be subject to information bias if some patient data were incomplete or inaccurately recorded. The potential for confounding variables, such as variations in post-operative care protocols or patient compliance with preoperative instructions, was also not fully explored, which may influence the findings.

Fifthly, this study did not include long-term post-operative outcomes, such as readmission rates or complications after discharge, which could provide a more comprehensive view of the factors affecting recovery and healthcare utilisation.

By acknowledging these limitations, future research can focus on addressing these gaps to further refine perioperative care and improve patient outcomes in a South African setting.

Future research should expand beyond a single private preoperative clinic to include public hospitals and clinics in various regions of South Africa. This would improve the generalisability of findings by capturing diverse patient populations and resource constraints that may influence perioperative outcomes.

Increasing the sample size, especially in underrepresented subgroups such as patients with renal dysfunction or those undergoing specific surgeries, would improve statistical power. This would allow for more robust analyses of associations between risk factors (e.g. ASA grading and RCRI) and outcomes like LOS. Multi-centre studies could provide a larger, more varied cohort for better insights.

Research should delve deeper into confounding factors such as variations in post-operative care protocols, patient adherence to preoperative instructions and social determinants of health. Understanding these influences could help refine interventions to reduce variability in patient outcomes.

Given the unique healthcare challenges in South Africa, future studies should aim to identify strategies for optimising perioperative care tailored to the resource availability and population needs of both private and public healthcare sectors.
